# The role of C-terminus carbohydrate-binding domain of *Vibrio cholerae* haemolysin/cytolysin in the conversion of the pre-pore β-barrel oligomer to a functional diffusion channel

**Published:** 2011-02

**Authors:** Budhaditya Mazumdar, Sreerupa Ganguly, Amar N. Ghosh, Kalyan K. Banerjee

**Affiliations:** *Division of Biochemistry, National Institute of Cholera & Enteric Diseases (ICMR), Kolkata, India*; * *Division of Electron Microscopy, National Institute of Cholera & Enteric Diseases (ICMR), Kolkata, India*

**Keywords:** Haemolysin, Lectin domain, Lipid bilayer, Pore-formation, *V. cholerae*

## Abstract

**Background & objectives:**

*Vibrio cholerae* cytolysin/hemolysin (VCC) is a 65 kDa pore-forming toxin (PFT) secreted by O1 El Tor and non-O1 strains. The purified toxin, which contains two C-terminus carbohydrate-binding domains in addition to the cytolytic domain at the core, causes lysis of a wide spectrum of eukaryotic cells at picomolar concentrations, apoptogenesis of intestinal and immune cells and accumulation of fluid in rabbit ligated ileal loop. Therefore, it may potentially complement the action of cholera toxin (CT) in diarrheagenic strains that do not produce CT. We showed earlier that β1-galactosyl-terminated glycoconjugates are strong inhibitors of its pore-forming activity, though carbohydrates are not functional receptors of VCC. Here, we investigate how the 15 kDa C-terminus β-prism lectin domain contributed to pore formation in erthrocytes.

**Methods:**

VCC was isolated from the culture supernatant of late log phase grown bacteria and purified to homogeneity by chromatography. The 50 kDa truncated variant was generated by restricted proteolysis. Liposome was prepared by sonication of a suspension of phospholipids and calceine release assay was done by spectrofluorometric monitoring of the released dye trapped in liposome. Formation of β-barrel oligomers in erythrocyte stroma was monitored by scanning electron microscopy.

**Results:**

Proteolytic truncation of the C-terminus β-prism lectin domain decreased hemolytic activity of the toxin by ~800-fold without causing a significant change in pore-forming activity toward synthetic lipid vesicles devoid of incorporated glycoproteins/glycolipids. Truncation at the C-terminus did not impair membrane-binding or assembly to the oligomeric pore.

**Interpretation & conclusions:**

Our data indicated that the C-terminus domain played a critical role in translocation of the pre-pore oligomeric assembly from the cell surface or lipid-water interface to the hydrocarbon core of the membrane bilayer, signaling the formation of functional diffusion channels.

*Vibrio cholerae* cytolysin/haemolysin (VCC)^1^ is a prominent member of a large, heterologous family of membrane-damaging proteins, collectively called pore-forming toxins (PFTs)^2^,^3^ that cause lysis of the target cell by punching transmembrane holes in the plasma membrane lipid bilayer. VCC is exported by the majority of *V. cholerae* O1 and non-O1 strains to the culture supernatant as the 79 kDa precursor, prohaemolysin, which on proteolytic removal of the N-terminal 15 kDa pro-domain converts to the fully mature 65 kDa toxin^4^. The purified toxin monomer binds to biological and synthetic membranes at picomolar concentrations and transforms eventually into a bilayer-inserted heptameric channel of internal diameter ~1.5 nm that mediates diffusion of small molecules and ions leading eventually to swelling and colloid osmotic lysis^5^. VCC acts on a wide spectrum of eukaryotic cells including erythrocytes, enterocytes and lymphocytes evoking responses that depend on the cell type. Specifically, the purified toxin stimulates fluid secretion in rabbit ligated ileal loop and causes diarrhoea leading to death of suckling mice^6^. It causes death of human intestinal cells, presumably by irreversible depletion of ATP^7^ and extensive vacuolation and eventually death of Hela and Vero cells^8^. VCC causes apoptosis of intestinal cells^9^ and induces caspase 9-dependent apoptogenesis of mouse peritoneal B-1a cells involved in IgA production, presumably by stimulating the mitochondrial pathway of apoptosis^10^. These data suggest that VCC is endowed with a spectrum of deleterious activities toward eukaryotic cells though its precise role in pathogenesis of cholera remains somewhat unclear.

Apart from the cellular responses evoked by PFTs that might make these critical to pathogenesis of the respective disease, PFTs are objects of considerable interest to the structural biologist and biochemist^2^,^3^. In contrast to proteins which usually exist in one of two incompatible conformations associated with water-soluble and integral membrane proteins, PFTs show dimorphism in existing initially as water-soluble monomers and terminally as β-barrel transmembrane oligomeric pores in the functionally active stage. The two states are separated by at least three biochemical events, *viz*., interaction of the toxin monomer with target membranes, self-assembly on the cell surface by circular oligomerization and insertion into the lipid bilayer core^2^,^3^. Further, the β-barrel oligomer assembled on the cell surface translocates spontaneously to the hydrocarbon core of the membrane bilayer without recruiting other proteins or energy transducers^11^. The molecular basis of how a PFT manages to exist in two states^12^ or move from water to the lipid bilayer core is not clear.

The three-dimensional structure of the 79 kDa cytolytically inactive precursor of the toxin, pro-VCC at 2.3Ǻ resolution reveals several distinct domains in the protein that distinguish it from other small and large β-barrel PFTs^13^. In addition to the core cytolysin domain involved in assembly and membrane-spanning, VCC has an N-terminal pro-domain that locks the oligomerization domain in an inactive conformation and two contiguous lectin domains at the C-terminus^13^. Specific affinity of VCC for β1-galactosyl-terminated glycoconjugates, apparently mediated by the C-terminus 15 kDa β-prism lectin domain homologous to the sugar-binding domain of the plant lectin jacalin^13^, was first reported from this laboratory^14^. However, the sensitivity of rabbit erythrocytes to VCC was found to be inversely correlated with the surface density of β1-galactosyl moiety, suggesting that the carbohydrate-dependent interaction of the toxin with the target membrane is unlikely to be the primary event in pore-formation^14^. Interestingly, the surface hydrophobicity of the VCC monomer is unusually high for a water-soluble protein and as a result, the toxin indulges in detergent-like interaction with synthetic and biological membranes in absence of specific receptors^15^.

Though not essential for membrane-binding, the carbohydrate-binding domains of VCC seem to play a significant role in generation of a functional channel in the target membrane^16^. Proteolytic removal of the β-prism lectin domain causes ~1000-fold reduction in specific haemolytic activity. Phosphatidylcholine-cholesterol vesicles devoid of protein and carbohydrate avidly take up VCC from water and induce oligomerization, but are 1000-fold less susceptible than erythrocytes to toxin. Recent cryo-electron microscopic analysis of the heptamers of the 65 kDa holotoxin and the 50 kDa truncated variant of VCC lacking the β-prism lectin domain (hereinafter referred to as VCC50) revealed significant differences in the shape and symmetry of the two oligomers^17^. These data suggest that the lectin domain plays a critical role in generation of a functional channel by an as yet obscure mechanism. In the present report, we demonstrate that there is no significant change in the qualitative features of interaction of VCC with liposome, *e.g*., partial unfolding of the toxin at the lipid-water interface^15^ and self-assembly to the oligomeric pore as a result of the truncation at the C-terminus. Since there is a dramatic decrease in efficiency of permeabilization of the membrane by VCC50 relative to the holotoxin, it is possible that truncation of VCC at the C-terminus end abrogates the pore-formation process at the pre-pore assembly stage.

## Material & Methods

The purification and biochemical characterization of VCC was carried out at the Division of Biochemistry and the Electron Microscopy at the Division of Electron Microscopy, National Institute of Cholera and Enteric Diseases, Kolkata.

### 

#### Isolation and purification of VCC:

VCC was purified to homogeneity as described earlier^14^, with some modification. *V. cholerae* non-O1 strain V_2_, a clinical isolate, a generous gift of R. Sakazaki, Tokyo, Japan was grown to stationary phase (~7 h) in Brain Heart Infusion (BHI, Becton-Dickinson, USA) broth at 37°C with shaking. Bacteria were removed by centrifugation at 18 000 × g and the supernatant was passed through a DEAE-cellulose column in 25 mM sodium phosphate buffer containing 3 mM NaN_3_ and 1 mM EDTA, *p*H 7.2 (Buffer A). The 79 kDa haemolytically inactive pro-VCC, which eluted unretarded leaving LPS and lipid vesicles bound to the column, adsorbed quantitatively to a phenyl-Sepharose CL-4B column in buffer A and was subsequently desorbed by 60 per cent ethylene glycol. The crude pro-toxin was subjected to anion exchange chromatography on PBE-94 Polybuffer Exchanger-94 (30 × 1 cm; Amersham, UK) and hydrophobic interaction chromatography on phenyl-Sepharose CL-4B column (20 × 0.75 cm). The pure protoxin was eluted by a linear 0-80 per cent ethylene glycol gradient and converted to the 65 kDa mature VCC by passing through a column of bovine trypsin immobilized to Sepharose CL-4B (Sigma, USA) in 100 mM Tris-HCl buffer containing 10 mM CaCl_2_, *p*H 7.8. The purified VCC migrated as a single polypeptide with M_r_ 65 kDa in SDS-polyacrylamide gel electrophoresis^18^ (SDS-PAGE) and caused 50 per cent haemolysis of a 1 per cent (vol/vol) suspension of rabbit erythrocytes in phosphate-buffered saline (PBS) in 1 h at 25°C at 100 pM concentration (~ 6 ng/ml). The haemolytic activity was assayed spectrophotometrically by monitoring the decrease in turbidity of a suspension of erythrocytes at 600 nm.

#### Preparation of the 50 kDa variant, VCC50:

VCC is resistant to proteolysis by trypsin under native condition^15^. To remove the 15 kDa β-prism lectin domain, we incubated VCC with trypsin at an enzyme: substrate ratio of 1:100 in 1.7 M urea in 100 mM Tris-HCl, 10 mM CaCl_2_. We demonstrated previously^12^ that there is no global unfolding of VCC at <2 M urea although the β-prism lectin domain was rendered susceptible to proteolysis by trypsin under the same condition^15^. Urea was dialyzed out and contaminating traces of the undigested 65 kDa toxin were removed by chromatography.

#### Analytical methods:

Protein was estimated by the modified Folin-Ciocalteu method^19^ using bovine serum as a standard and neutral sugar by the phenol-sulphuric acid method^20^ using D-glucose as a standard. Cholesterol in the erythrocyte stroma was estimated enzymatically by using cholesterol oxidase and the H_2_O_2_ generated was quantified colorimetrically by using a mixture of peroxidase, phenol and 4-aminophenazone, as described by the manufacturer (Chemelex, S.A. Barcelona, Spain).

#### Liposome preparation and calceine release assay:

A mixture of phosphatidylcholine and cholesterol (1:1 by wt) in chloroform-methanol (2:1 by vol) was evaporated to dryness under reduced pressure and dispersed in PBS and subjected to sonication for 10 min with pulses of 30 sec and 1 min rest at 23 kHz. The multilamellar vesicles were discarded by centrifugation at 30 000xg. Large unilamellar vesicles, separated from small unilamellar ones by size exclusion chromatography on Sepharose CL-4B in PBS, were used for further work. For preparation of liposomes in which calceine (2^/^,7^/^-bis-[N,N,-bis-(carboxymethyl)-aminomethyl]-fluorescein) was trapped, the dried film of lipid was hydrated in PBS containing 5 mM calceine, followed by processing as above. Free calceine not incorporated into the liposome was removed by gel filtration on Sephadex G-25. Permeabilization of the liposomal membrane was assayed by incubating the liposome with VCC for varying lengths of time and quantifying the released calceine in a spectrofluorimeter (excitation wavelength: 485 nm; emission wavelength: 520 nm). Fluorescence intensity corresponding to the lysis of liposome in 1 per cent (vol/vol) Triton X-100 was taken as equivalent to 100 per cent lysis.

#### Transmission electron microscopy:

Liposomes and rabbit erythrocyte stroma, prepared by hypotonic lysis of PBS-washed red blood cells were incubated with VCC and VCC50 at room temperature, washed and negatively stained with 2 per cent uranyl acetate and examined with a FEI Tecnai 12 BioTwin Transmission Electron Microscope (Netherlands).

## Results & Discussion

### 

#### Truncation at the C-terminus causes a dramatic decrease in pore-forming activity toward erythrocytes but not toward liposome:

**Fig. 1:**
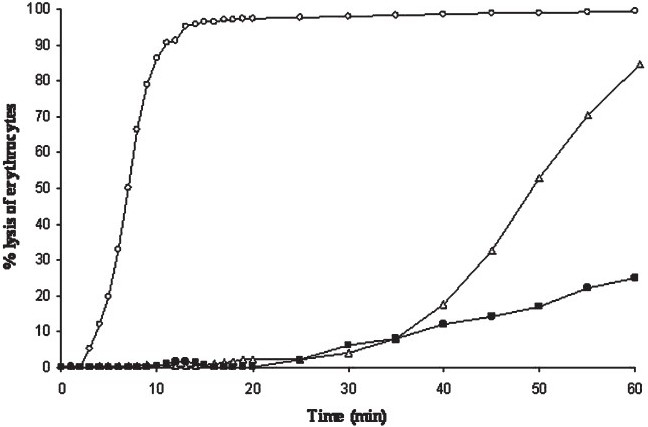
Kinetics of haemolysis of rabbit (○), sheep (∆) and human (■) erythrocytes (1% vol/vol) at a VCC concentration of 10 ng/ml. Lysis was monitored spectrophotometrically by measuring the decrease in A_600_ with time. Single experimental values are representatives of at least 5 determinations with different batches of vcc.

The specific haemolytic activity, defined as the lowest concentration of the toxin causing 50 per cent haemolysis of a 1 per cent suspension of erythrocytes at 25°C in 1 h, of purified VCC toward rabbit, sheep and human erythrocytes was in the range of 100-400 picomolar. However, the three erythrocytes differed sharply in the kinetics of haemolysis, with the rabbit cells being the most susceptible (Fig. 1). Because membrane-binding and oligomerization are essentially complete within 2 min of incubation^15^, it is possible that secondary membrane events like bilayer-insertion^2^,^3^ were rate-limiting in haemolysis. Proteolytic removal of the β-prism carbohydrate-binding domain led to an 800-fold decrease in haemolytic activity toward rabbit erythrocytes. When phosphatidylcholine-cholesterol liposomes with entrapped calceine were incubated with the two toxin variants and permeabilization of the bilayer was monitored by assaying for the concentration of the leaked probe spectrofluorimetrically, there was no difference between VCC and VCC50, but the vesicles were found to be about 1000-fold less sensitive than erythrocytes (data not shown). Collectively, these data indicated that presence of the lectin domain of VCC augmented pore-forming activity to erythrocytes by three orders of magnitude without having a similar effect on synthetic vesicles. It seems likely that interaction of the toxin with a specific membrane glycoprotein/glycolipid component, though not absolutely essential for perforating the lipid bilayer, increased the efficiency of the process dramatically. As expected, absence of the lectin domain had no effect on permeabilization of the lipid bilayer of synthetic vesicles which lacked glycocojugates.

#### Interactions of VCC and VCC50 with synthetic lipid vesicles follow similar mechanism:

**Fig. 2:**
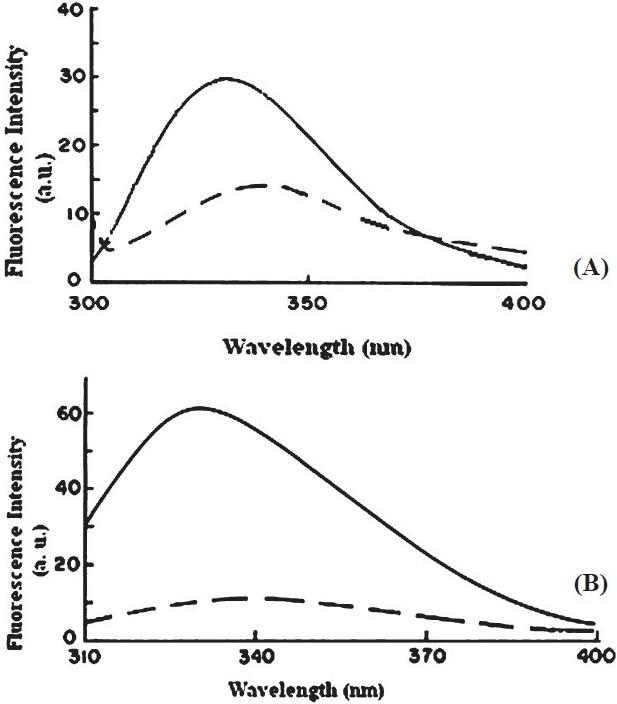
Lipid-induced perturbation Trp fluorescence emission spectrum of VCC (A) and VCC50 (B). The two cytolysin forms were incubated with phosphatidylcholine-cholesterol liposome at a protein concentration of 50 µg/ml and lipid:protein ratio of 1:10. Samples were excited at 295 nm with slit widths set at 2.5 nm.

We reported earlier that movement of VCC to the lipid-water interface was accompanied by disruption of the native toxin structure^15^. To see if VCC50 exhibited similar feature in its primary interaction with the lipid bilayer, we incubated the two toxins with liposome and monitored conformational change by tryptophan intrinsic fluorescence (Figs. 2A and B). It is seen that the red shift in λ_max_ as well as quenching fluorescence of VCC50 was similar to that observed with VCC, indicating that the truncated variant showed a similar propensity to interact with the bilayer and to move into the lipid-water interface with partial melting of the native structure.

**Fig. 3:**
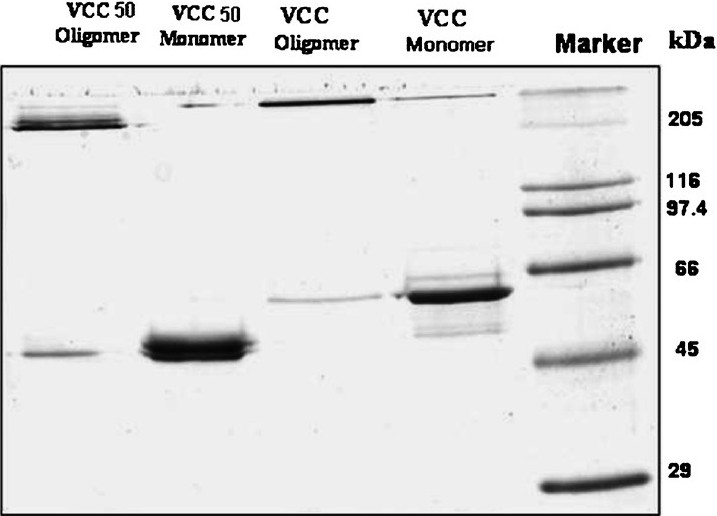
SDS-PAGE analysis of VCC and VCC50 monomer and oligomer. Proteins were run in 10 per cent polyacrylamide gel and stained in Coomassie Brilliant Blue.

**Fig. 4:**
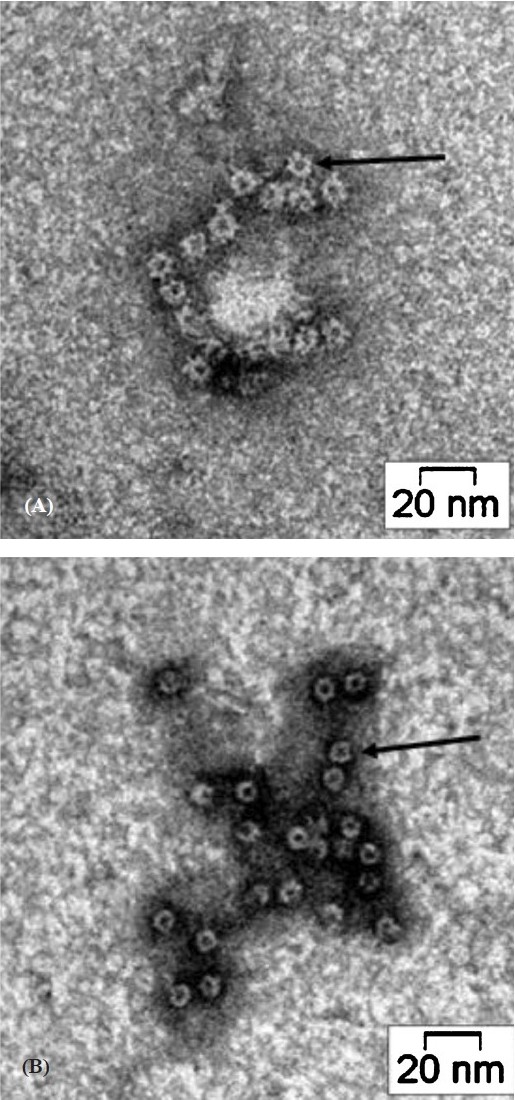
Transmission electron micrographs of VCC (A) and VCC50 (B) on rabbit erythrocyte stroma. Following incubation, unbound toxins were washed out and the stroma were negatively stained with 2 per cent uranyl acetate and examined with a FEI Tecnai 12 BioTwin Transmission Electron Microscope. The β- barrel pores are marked by arrow

Next, we wanted to ascertain whether the two toxin variants formed oligomers in contact with lipid vesicles that have the same stoichiometry and similar morphology. On SDS-PAGE analysis (Fig. 3), both the toxins are seen to form heptamers in contact with liposomes. Further, the electron micrographs of rabbit erythrocyte stroma incubated with VCC and VCC50 (Fig. 4) showed the membrane surface to be decorated with ring-like oligomeric pores surrounding a central hole. Notably, the dimensions of the pores generated by the two toxin variants were of similar magnitude. It seems, therefore, that the impairment in pore-forming activity as a consequence of C-terminus deletion was not due to a difference in primary interaction with the membrane or toxin self-assembly.

#### VCC but not VCC50 gets anchored to the erythrocyte membrane cytoskeleton:

**Fig. 5:**
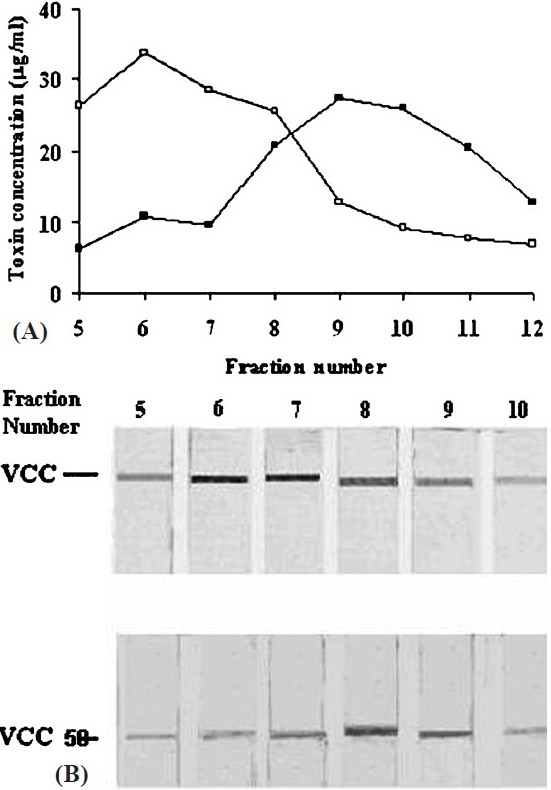
Size distribution of VCC and VCC50 micelles with rabbit erythrocyte membrane components and Triton X-100. Following incubation of the toxin with stroma at 25°C, the free toxins were removed by microfuging and the mixture was dispersed in 1 per cent Triton X-100 at 4°C. The suspension was fractionated on Sepharose CL-4B (30 × 0.7 cm) at 4° C. Fractions of 1 ml were collected and quantified for VCC (○) and VCC50 (●) by ELISA (A). Immunoblots of the fractions developed with rabbit anti-VCC antibody and goat anti-rabbit IgG conjugated to alkaline phosphatase are also shown (B).

Since similar interaction of VCC and VCC50 with liposome was correlated with their comparable pore-forming activity toward these vesicles, we examined if interaction of the two toxin forms could be biochemically distinguished. The complex of VCC and VCC50 with rabbit erythrocyte stroma were dispersed in 1 per cent Triton X-100 at 4°C and subjected to gel filtration on Sepharose CL-4B. Fractions were assayed for VCC by ELISA (Fig. 5A) and also by Western immunoblot^21^ (Fig. 5B). To see if the two toxin forms showed a tendency to associate with distinct membrane bilayer domains such as lipid rafts^22^, the fractions were also monitored for both cholesterol and neutral sugars (data not shown). We found that the larger micelles, made detergent-insoluble by association with the erythrocyte cytoskeleton, were preferentially enriched in VCC while the smaller micelles in the truncated variant. Interestingly, insertion of VCC into the membrane bilayer did not cause any redistribution of the lipid raft components like cholesterol and glycolipids implying that raft association was not critical for pore-formation by VCC. It is known that aerolysin, a PFT from *Aeromonas hydrophila* with structural and functional similarity to VCC, depends critically for its association with lipid rafts for insertion into the bilayer to generate a functional channel^23^.

In conclusion removal of the β-prism lectin domain causes a dramatic decrease in specific haemolytic activity of VCC though there is neither any gross change in biophysical characteristics nor any significant impairment in membrane-binding or oligomerization potential. So, it is tempting to speculate that the truncated variant of the toxin, VCC50 adopts a conformation that is less competent than the 65 kDa holotoxin to insert into the bilayer core. The molecular mechanism of how anchoring of the toxin to membrane glycoconjugates promotes its translocation from the target cell surface to the hydrophobic core of the bilayer leading eventually to perforation of the bilayer remains to be elucidated.
